# Improved anti-tumor efficacy via combination of oxaliplatin and fibrin glue in colorectal cancer

**DOI:** 10.18632/oncotarget.23507

**Published:** 2017-12-20

**Authors:** Yuzhu Hu, Ting Yu, Xiaoxiao Liu, Yihong He, Lihong Deng, Jiajuan Guo, Yuanqi Hua, Ting Luo, Xiang Gao

**Affiliations:** ^1^ Department of Head & Neck and Mammary Oncology and Department of Medical Oncology, Cancer Center, State Key Laboratory of Biotherapy, Laboratory of Molecular Diagnosis of Cancer, West China Hospital, West China Medical School, Sichuan University, Chengdu 610041, PR China; ^2^ Department of Neurosurgery and Institute of Neurosurgery, State Key Laboratory of Biotherapy/Collaborative Innovation Center for Biotherapy, West China Hospital, West China Medical School, Sichuan University, Chengdu 610041, PR China

**Keywords:** colorectal cancer, oxaliplatin, fibrin glue, anti-tumor activity, cell proliferation

## Abstract

Colorectal cancer is very common worldwide and advanced colorectal cancer exhibited very poor clinical outcome. Oxaliplatin (OXP) is one of the principal chemotherapeutic agents in colorectal cancer treatment presenting impressive anti-tumor ability, limited by adverse effect in clinical practice. Fibrin glue (FG) is a biocompatible formulation made of fibrinogen and thrombin, extensively used in surgery for hemostasis, tissue adhesion and sealing. In this study, FG was innovatively applied as OXP delivery system and results showed enhanced anti-tumor performance in subcutaneous model and abdominal metastasis model of murine colorectal cancer compared with that of OXP used alone. It is revealed that combination of OXP and FG could increase activated CD8^+^ T cells, reduce regulatory T (Treg) cells and increase interferon-γ (IFN-γ). Furthermore, results showed promoted tumor apoptosis, decreased proliferation and inhibited tumor angiogenesis by OXP and FG combination. No obvious systemic toxicity was observed in this study. Finally, our findings provided basis for promising application of OXP and FG combination in colorectal cancer treatment.

## INTRODUCTION

Colorectal cancer was one of the top three cancer types worldwide according to the Global Cancer Statistics in 2012 [1], which has become a heavy health burden. It was estimated that 49,190 deaths were caused by colorectal cancer in United States in 2016 [2]. Incidence rate of colorectal cancer in developed countries was several times higher than that in developing countries [1], however, the rising trend in some emerging developing countries due to popularization of westernized life style cannot be ignored [2–5]. With promoted preventive measures and advanced screening technology, colorectal cancer mortality rate was reported to decline in many countries [6, 7]. Nonetheless, 40 to 50 percent of colorectal cancer patients will suffer from recurrence or death after surgical resection, so further adjuvant treatment was needed [8].

Chemotherapy is the major treatment approach after surgery. For example, Fluorouracil plus Leucovorin (FU+LV) is the standard chemotherapy regimen for colon cancer patients [9, 10]. Oxaliplatin (OXP) is the third generation platinum derivatives with a significant role in colorectal management, which can inhibit DNA synthesis and replication and thus to decrease tumor cell growth. Many clinical trials have proved the addition of OXP in standard FU+LV chemotherapy (FOLFOX regimen) presented notable improved treatment efficiency in stage III and stage IV colon cancers, but its benefit in high-risk stage II colon cancers patients is yet to be confirmed [11–16]. However, toxicities are main concerns in use of OXP [17]. Therefore an ideal drug delivery vesicle is required to achieve slow and sustained release of OXP so as to reduce side effects without decreasing efficacy.

Fibrin glue (FG) is a medical hemostat made of fibrinogen and thrombin, which is commonly used in surgical procedures, by formation of fibrin clot thereby promoting hemostasis, tissue adhesion and sealing. FG exhibits several advantages such as good biocompatibility, safety and biodegradability. FG was proved to be a desirable vesicle for antibiotics, drugs, genes and growth factors [18–23]. Moreover, several studies have demonstrated combination of FG and anti-tumor agent as drug delivery system, showing inspiring results in chemotherapy of pancreatic cancer, retinoblastoma, liver cancer, melanoma, gastric cancer, esophageal cancer and glioma [24–30]. In spite of this, few research has reported FG application in colorectal cancer chemotherapy, thus it will be intriguing to combine OXP and FG in colorectal cancer treatment.

In our study, we firstly used FG as sustainable and safe drug delivery system for OXP in both subcutaneous model and abdominal metastasis model of murine colon cancer. Moreover, we further innovatively explored the effect of OXP and FG in immune microenvironment, tumor apoptosis, tumor proliferation and tumor angiogenesis, aiming at providing evidence for novel chemotherapy approach in the management of colorectal cancer.

## RESULTS

### Inhibitory effect of OXP on colon tumor cells growth

**Figure 1:**
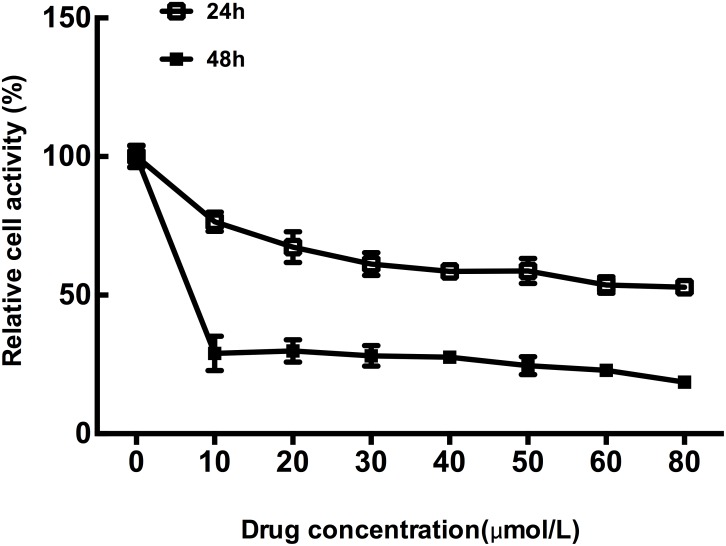
Inhibitory effect of oxaliplatin on colon tumor cells growth The cell survivals of CT26 cells exposed to different concentrations of Oxaliplatin for 24h and 48h were measured by MTT assay. Each point represented the mean ± standard error of mean (SEM), n=6.

MTT assays were performed in CT26 cells treated with different concentration of OXP for 24h and 48h, respectively, to verify the effect of OXP on colon cancer cells metabolic activity. Results showed that CT26 cells growth was significantly inhibited by OXP in concentration and time dependent manners (Figure 1). MTT assay results determined the inhibitory effect of OXP to colon tumor cells growth.

### *In vivo* antitumor activity

**Figure 2:**
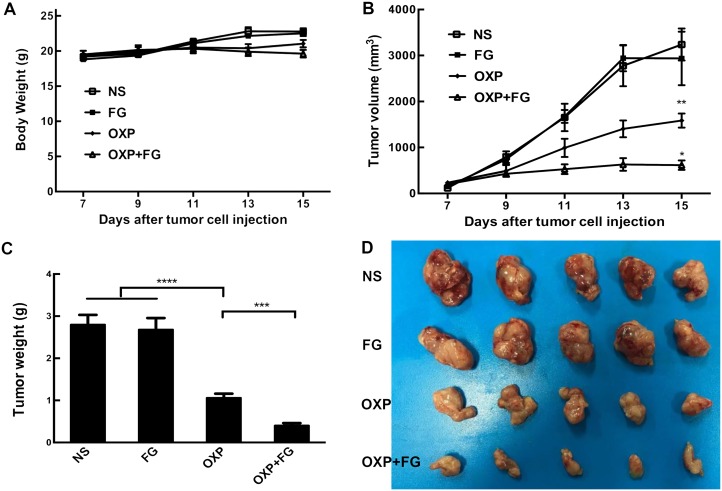
Oxaliplatin and FG combination showed enhanced anti-tumor effect in subcutaneous model of colon cancer Mice bearing colon tumor were randomly allocated into NS group, FG group, OXP group and OXP+FG group and started to receive treatment on 7^th^ day after inoculation. **(A)** Mice body weights of different treatment groups (P>0.05, OXP+FG versus NS group, FG group and OXP group). **(B)** Changes of subcutaneous tumor volumes of different groups (P<0.01, OXP group versus NS and FG groups. P<0.05, OXP+FG group versus OXP group). **(C)** Tumor weights of different groups (P<0.0001, OXP group versus NS and FG groups. P<0.001, OXP+FG group versus OXP group). **(D)** Photograph of tumors on the 15^th^ day after implantation. Each bar represented the SEM, n=5.

**Figure 3:**
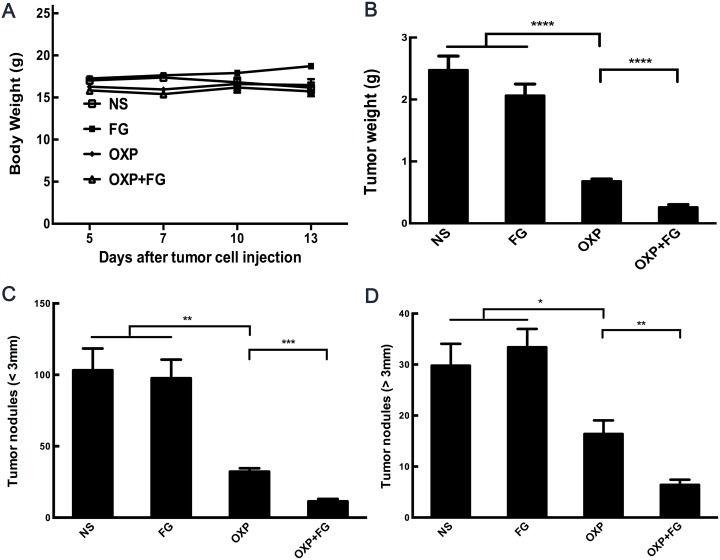
Oxaliplatin and FG combination showed enhanced anti-tumor effect in abdominal metastasis model of colon cancer Mice bearing colon tumor received different treatments were divided into four groups: NS group, FG group, OXP group and OXP+FG group and started to receive treatment on 5^th^ day after peritoneal injection. **(A)** Mice body weights of different treatment groups (P<0.05, OXP+FG versus NS group, FG group and OXP group). **(B)** Tumor weights of different groups (P<0.0001, OXP group versus NS and FG groups. P<0.0001, OXP+FG group versus OXP group). **(C)** Tumor nodules amount (<3mm) (P<0.01, OXP group versus NS and FG groups. P<0.001, OXP+FG group versus OXP group). **(D)** Tumor nodules amount (>3mm) (P<0.05, OXP group versus NS and FG groups. P<0.01, OXP+FG group versus OXP group). Each bar represented the SEM, n=5.

Subcutaneous mouse model and abdominal metastasis mouse model were established to evaluate antitumor effect of OXP+FG in colon cancer. Mice bearing colon cancer were randomly allocated into Normal Saline (NS), FG, OXP and OXP in combination with FG (OXP+FG) groups in both models. In subcutaneous model, there was no obvious difference among body weights of NS group, FG group, OXP group and OXP+FG group (Figure 2A). Subcutaneous tumor volumes were dramatically smaller in OXP+FG group compared with those in NS group, FG group and OXP group (Figure 2B). Consistently, subcutaneous tumor weights of OXP+FG group were distinctly suppressed compared with other treatment groups (Figure 2C). Images of subcutaneous tumor were shown (Figure 2D). In abdominal metastasis model, body weights of OXP and FG group were slightly less than body weights of other three groups (Figure 3A). In addition, OXP+FG group showed reduced ascites compared with NS group, FG group and OXP group (data not shown). Tumor weights of OXP+FG group were significantly less versus those of NS group, FG group and OXP group (Figure 3B). Furthermore, there was a significant decrease of nodules amount (<3mm and >3mm) in OXP+FG group compared with that in other groups (Figure 3C and 3D). In summary, all results above proved that OXP+FG treatment showed enhanced anti-tumor activity in both subcutaneous models and abdominal metastasis model of colorectal cancer, in comparison with NS, FG and OXP treatments.

### OXP+FG increased activated CD8^+^ T cells and reduced Treg cells *in vivo*

**Figure 4:**
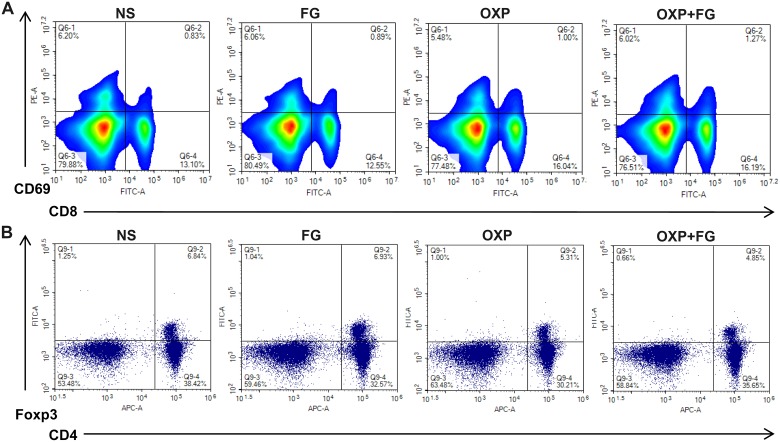
OXP and FG combination promoted activated CD8^+^ T cells and reduced regulatory T cells Lymphocytes from mice spleens were stained with CD8, CD69, CD4 and Foxp3 fluorochrome-labeling antibodies. Cells with positive CD8 and CD69 staining indicated activated CD8^+^ T cells. Cells with positive CD4 and Foxp3 indicated regulatory T cells (Treg) cells. **(A)** Representative images of flow cytometry result for activated CD8^+^ T cells in different treatment groups. **(B)** Representative images of flow cytometry result for Treg cells in different treatment groups.

To explore the anti-tumor mechanism of OXP+FG, analysis of immune microenvironment in mice was carried out in our study. Interestingly, activated CD8^+^ T cells percentage was found increased while Treg cells percentage was decreased in mice spleens of OXP+FG group, in comparison of NS group, FG group and OXP group (Figure 4A and 4B). The results demonstrated that OXP+FG could increase activated CD8^+^ T cells and reduce Treg cells *in vivo*.

### OXP+FG increased IFN-γ concentration

**Figure 5:**
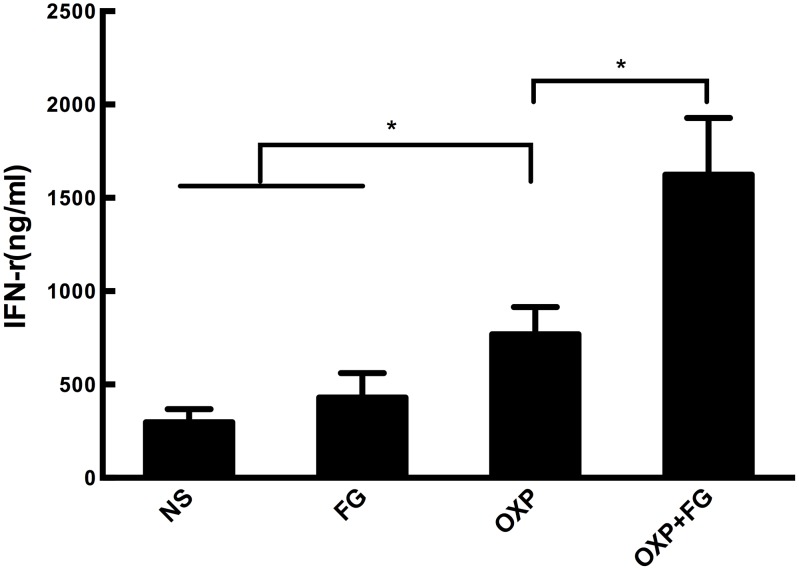
OXP and FG combination significantly increased INF-γ concentration IFN-γ in ascites of mice was detected with ELISA method. Significantly increased concentration of IFN-γ was observed in OXP group compared with NS group, FG group (P<0.05, OXP group versus NS and FG groups). The concentration of IFN-γ in OXP+FG group was higher than that of OXP group (P<0.05, OXP+FG group versus OXP group). Each bar represented the SEM, n=5.

To determine the quantity of IFN-γ in ascites, an IFN-γ ELISA Kit was applied in our study. Results showed that concentration of IFN-γ in ascites of OXP+FG group was significantly higher than that in ascites of NS group, FG group and OXP group (Figure 5). Therefore, results suggested that OXP+FG evoked more IFN-γ against tumor cells.

### OXP+FG promoted tumor apoptosis *in vivo*

**Figure 6:**
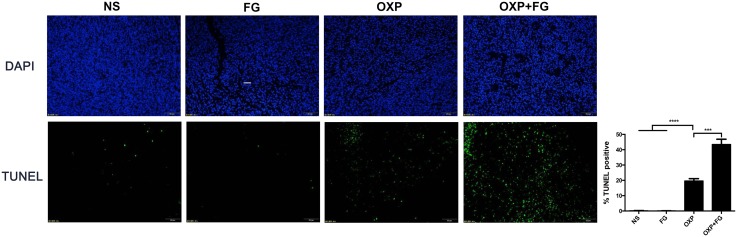
OXP and FG combination promoted tumor apoptosis *in vivo* TUNEL assay of tumor sections was performed to analyze tumor apoptosis. Cells with positive TUNEL staining represented apoptotic cell. Results demonstrated increased apoptotic tumor cells in OXP group compared with NS group and FG group (P<0.0001, OXP group versus NS and FG groups). There were more apoptotic tumor cells in OXP+FG group than that in OXP group (P<0.001, OXP+FG group versus OXP group). Representative images were showed above. Each bar represented the SEM, n=5.

TUNEL assay was performed to investigate tumor apoptosis in different treatment groups. Tumor cells with positive TUNEL staining represented apoptotic tumor cells. In OXP+FG group and OXP group, TUNEL staining positive cells were obviously more than those in NS group and FG group. Moreover, TUNEL staining positive cells in OXP+FG group were more than OXP group (Figure 6). Therefore, results suggested OXP+FG treatment promoted more tumor apoptosis compared with NS, FG and OXP treatment alone.

### OXP+FG decreased tumor proliferation *in vivo*

**Figure 7:**
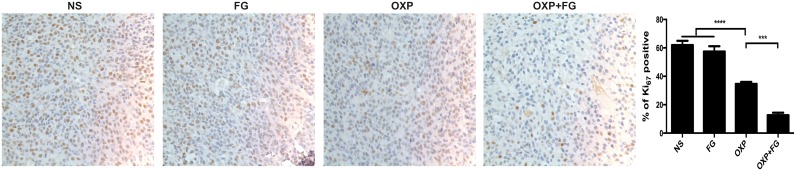
OXP and FG combination attenuated tumor proliferation *in vivo* Ki_67_ staining of tumor sections was applied to explore tumor proliferation. Cells with positive Ki_67_ staining represented cells with high proliferation activity. Results showed decreased proliferation of tumor cells in OXP group, compared with that in NS group and FG group (P<0.0001, OXP group versus NS and FG groups). In OXP+FG group, there were less tumor cells with high proliferation activity than that in OXP group (P<0.001, OXP+FG group versus OXP group). Representative images of each group were showed above.

Ki_67_ staining was carried out to detect tumor proliferation in NS group, FG group, OXP group and OXP+FG group. Ki_67_ staining positive cells indicated cells with high proliferation activity. In OXP+FG group and OXP group, Ki_67_ staining positive cells were fewer than those in NS group and FG group. Additionally, OXP+FG group showed less Ki_67_ staining positive cells than OXP group (Figure 7). Results demonstrated OXP+FG exhibited stronger inhibitory effect on tumor proliferation *in vivo*, compared with NS, FG and OXP treatment alone.

### OXP+FG inhibited tumor angiogenesis *in vivo*

**Figure 8:**
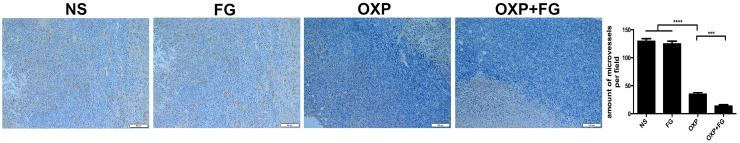
OXP and FG combination repressed tumor angiogenesis CD31 staining can be used to label microvessel in tumor sections. Results showed reduced microvessel densities (MVD) of tumor cells in OXP group, compared with that in NS group and FG group (P<0.0001, OXP group versus NS group and FG group.). There were less microvessels in OXP+FG group than that in OXP group (P<0.001, OXP+FG group versus OXP group). Representative images of each group were showed above.

As angiogenesis is an important event in tumor development and progression, investigation on microvessel density (MVD) of tumor sections was performed with CD31 staining method. In OXP+FG group and OXP group, immunohistochemical results showed decreased MVD compared with that in NS group and FG group. Moreover, there were less CD31 staining positive cells in OXP+FG group than those in OXP group (Figure 8). Results revealed that OXP+FG might play anti-tumor activity through suppressing angiogenesis of tumor.

### Safety assessment

**Figure 9:**
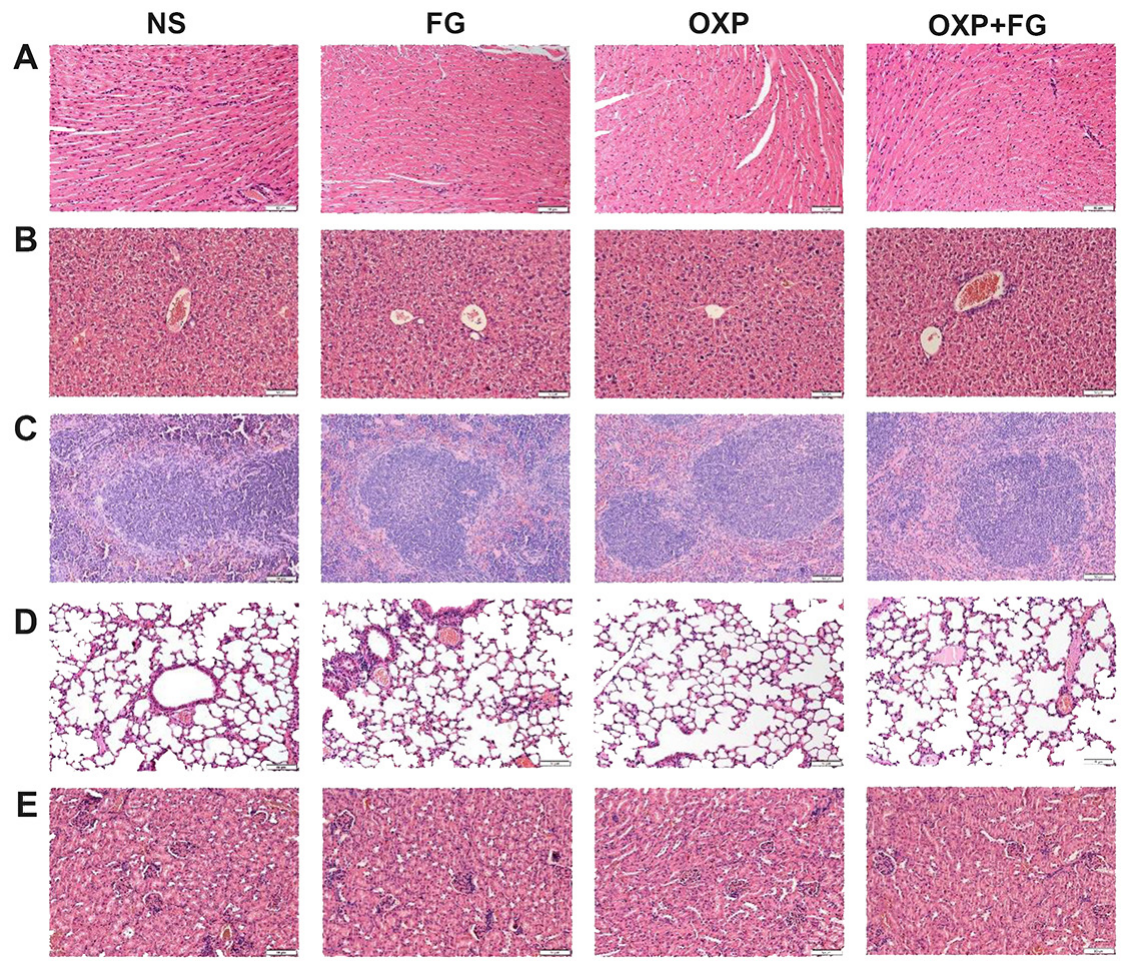
OXP and FG combination exhibited no toxicity to vital organs HE staining images of heart **(A)**, liver **(B)**, spleen **(C)**, lung **(D)** and kidney **(E)** were presented. No obvious pathohistological abnormalities were observed in NS group, FG group, OXP group and OXP+FG group.

**Figure 10:**
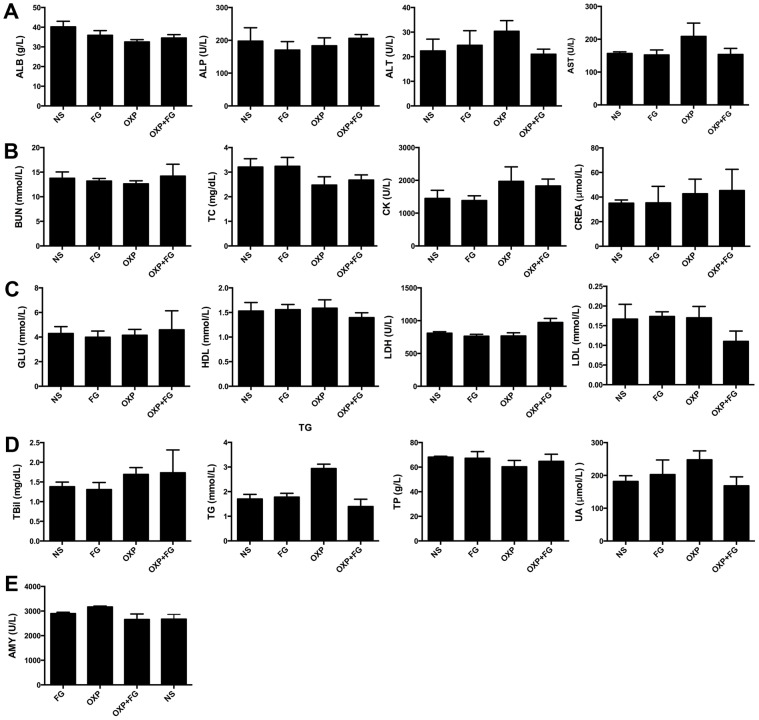
OXP and FG combination exhibited no systemic toxicity *in vivo* Blood serums of mice from different treatment groups were assessed for some biochemical parameters (ALB, albumin; ALP, alkaline phosphatase; ALT, alanine transaminase; AST, aspartate aminotransferase; BUN, blood urea nitrogen. TC, total cholesterol; CK, creatine kinase; CREA, creatinine; GLU, glucose; HDL, high density lipoprotein-cholesterol; LDH, lactate dehydrogenase; LDL, low density lipoprotein-cholesterol; TBil, total bilirubin; TG, triglycerides; TP, total protein; UA, uric acid; AMY, amylase). **(A)** ALB, ALP, ALT, AST (P>0.05, P>0.05, P>0.05, P>0.05 respectively, for all groups comparison). **(B)** BUN, TC, CK, CREA (P>0.05, P>0.05, P>0.05, P>0.05, respectively, for all groups comparison). **(C)** GLU, HDL, LDH, LDL (P>0.05, P>0.05, P>0.01, P>0.05, respectively, for all groups comparison). **(D)** TBil, TG, TP, UA (P>0.05, P<0.01, P>0.05, P>0.05, respectively, for all groups comparison). **(E)** AMY (P>0.05 for all groups comparison). Each bar represented the SEM, n=5.

To further evaluate the safety of FG and OXP combination on experiment animals, HE staining and serum biochemical parameters measurement were carried out. There was no visible treatment toxicity to vital organs according to the HE staining of heart, liver, spleen, lung and kidney sections (Figure 9). Test results of key biochemical parameters in serum indicated no differences among four treatment groups (Figure 10). Moreover, monitoring of animal growth status such as body weight, excretions and appearance showed no obvious abnormalities.

## DISCUSSION

Cancer has been a thorny issue for human health and colorectal cancer is among the most prevalent cancer types all around the world. Although great progresses were achieved on the management of colorectal cancer in recent decades, problems such as local recurrence and distal metastasis are urgent to be overcome. Chemotherapy regimen containing OXP shows pleasant anti-tumor performance but limited by drug adverse effects.

Our study innovatively combined OXP and FG in colon cancer treatment and achieved remarkable anti-tumor activity. Both murine colon cancer subcutaneous model and abdominal metastasis model were established for investigating the therapeutic effects and underlying mechanism. *In vitro*, OXP showed strong inhibitory effect to CT 26 cells growth. *In vivo*, OXP+FG presented remarkable enhanced anti-tumor effect in both subcutaneous model and abdominal metastasis model in comparison with NS, FG and OXP alone, through increasing activated CD8^+^ T cells, reducing Treg cells and evoking more IFN-γ. Moreover, OXP+FG suppressed tumor proliferation and tumor angiogenesis as well as induced tumor cell apoptosis. No obvious toxicity was exhibited *in vivo* among all treatment groups.

Some studies have reported enhanced anti-tumor performance of FG mixed with chemotherapeutic agents in a few cancers. Kitazawa monitored a sustained release of doxorubin (DOX) from FG containing sodium alginate in rats with liver cancer and reported its benefit in local delivery for higher drug concentration in tumor extracellular fluid than that in blood [27]. In addition, Ogura founded gemcitabine (GEM) and FG was more effective than GEM in inhibiting growth of human pancreatic cancer cells in nude mice [28]. Tanaka founded mixture of mitomycin (MMC) and FG showed twice the effect of MMC in gastric and esophageal cancer [25]. Our result was coincide with previous studies that chemotherapeutic agent OXP mixed in FG was more efficient in anti-tumor activity compared with OXP alone, which was probably due to maintained concentration and sustained release of drug. Moreover, no obvious systemic toxicity was observed in our study, demonstrating FG might be a desirable drug vesicle to reduce drug diffusion into distal normal tissue.

OXP is known to function through blocking the duplication of DNA. Recently, OXP was revealed to induce immunogenic cell death associated with High mobility group box 1(HMGB1) release and calreticulin exposure [31]. When in combination with liver-specific IL-12 or IL-7, OXP increased CD8^+^/ Treg cell ratio in murine metastatic colon cancer model, reducing immune suppressive response and further enhancing anti-tumor immune response [32–35]. Moreover, it was reported that colorectal cancer patients had increased percentage of Treg cells both in peripheral blood and tumor infiltrating lymphocytes [36]. Many present studies supported that T cells shifted from host-protective to tumor promoting Treg cells, associated with poor prognosis in various cancers [37–40]. Depletion of Treg cells improved anti-tumor immunity in colitis-associated colon cancer and improved the survival of post-surgery colorectal cancer patients [41, 42]. Our result showed suppressed Treg cells *in vivo* and enhanced anti-tumor activity by OXP and FG combination, which was consistent with their findings.

Activated CD8^+^ T cells are known as cytotoxic T cells and are very important in adaptive immunity, which can secret cytotoxic granules and induce target cell apoptosis thus to play anti-tumor role. However, CD8^+^ T cells are often hampered by Treg cells through cell contact and transforming growth factor (TGF)-β [43]. Our study found increased activated CD8^+^ T cells and reduced Treg cells along with enhanced anti-tumor performance in colorectal cancer and tumor cell apoptosis was promoted. Suzuki demonstrated intratumoral CD8^+^/Treg cell ratio was positively correlated with both disease-free survival and overall survival in colorectal cancer patients [44]. Our results provided supportive evidence to Suzuk's viewpoint.

IFN-γ is secreted by T helper (Th) 1 cells, CD8^+^ cytotoxic T cells, macrophages, NK cells and mucosal epithelial cells, playing versatile anti-tumor roles on cancer cells. IFN-γ participates in anti-proliferative, pro-apoptotic and anti-angiogenesis processes [45–48]. In addition, Ni founded IFN-γ showed synergistic effects with OXP to eliminate both colon cancer initiating label-retaining cells (LRCCs) and non-LRCCs [49]. Overacre-Delgoffe discovered IFN-γ could increase the fragility of Treg cells to heighten anti-tumor immunity [50]. In our study, we found there was a significantly increase of IFN-γ concentration in ascites of OXP+FG group compared with NS group, FG group and OXP group, which could be one of the reasons to enhance anti-tumor performance through promoting tumor cell apoptosis, suppressing angiogenesis and tumor cell proliferation.

In conclusion, our study innovatively proposed the combination of OXP and FG as a novel drug delivery system for colorectal cancer treatment. OXP+FG presented remarkablely enhanced anti-tumor efficacy in colorectal cancer in comparison with OXP alone, mediated by reducing immunosuppressive microenvironment, inhibiting tumor proliferation, attenuating angiogenesis and promoting tumor apoptosis. This is the first study to combine OXP and FG in chemotherapy of colorectal cancer and reveal its potential anti-tumor mechanisms. FG is now commonly used as medical hemostat for patients during surgery, thus it will be very meaningful and promising to realize local drug delivery by FG in chemotherapy of colorectal cancer patients.

## MATERIALS AND METHODS

### Reagents

Oxaplatin Injection (OXP) was obtained from Jiangsu Hengrui Medicine (China), Fibrin Glue (Human) was purchased from Shanghai RAAS (China). 3-(4, 5-dimethyl-2-thiazolyl)-2,5-diphenyl-2-H-tetrazolium bromide (MTT) was purchased from Sigma-Aldrich (USA) and dimethyl sulfoxide (DMSO) from KeLong Chemicals (China). Diaminobenzidine (DAB) was purchased from Servicebio (China). 4’, 6-diamidino-2-phenylindole (DAPI) was purchased from Beyotime (China). Antibodies purchased included: CD8, CD4, CD69 from Abcam (USA), goat anti-mouse Ki_67_ antibody, goat anti-mouse CD31 antibody and rabbit anti-goat horseradish peroxidase (HRP)-conjugated secondary antibody from Servicebio (China). Terminal deoxy-nucleotidyl transferase-mediated dUTP nick end labeling (TUNEL) kit was purchased from Promega (USA). Anti-human forkhead box P3 (Foxp3)/Transcription Factor Staining Buffer Set and mouse IFN-γ ELISA kit were purchased from affymetrix, eBioscience (USA).

### Cell lines

Colon carcinoma cell line CT 26 was purchased from American Type Culture Collection (ATCC Number: CRL-2638TM, USA). Cells were cultured in RPMI 1640 basal medium (Gibco, USA) supplemented with 10% fetal bovine serum (Gibco, USA), 0.1% Amikacin and incubated in plastic cell culture dishes in humidified incubator at 37°C with 5% CO2.

### MTT assay

Cells viability was measured by MTT assay. 3^*^10^3^ cells/well and 5^*^10^3^ cells/well were seeded into two 96-well plates, respectively, and incubated overnight. Then the culture medium was replaced by 200μl RPMI 1640 medium containing OXP at concentration of 0-80μg/ml with 6 duplicate wells for each concentration. After 24h and 48h incubation, 20μl MTT solution (5mg/ml) was added to each well and co-cultured with cells in dark humidified incubator at 37°C with 5% CO_2_ for 3 hours. Next, culture medium was removed and formazan was dissolved by 150μl DMSO for 10 minutes. Optical density (OD) was determined by spectrophotometer at 570nm with a microplate reader (OPTI max, Molecular Dynamics). Cell viability was expressed as: cell viability (%) = (mean OD in test wells) / (mean OD in control wells) ×100.

### Animal model establishment and treatment

6-8 weeks female BALB/c mice were obtained from the Animal Center Laboratory of Beijing HFK Bioscience (Beijing, China). All animals are provided with sterilized water and chow and maintained under SPF (specific pathogen free) environment. All study procedures were approved by the Animal Ethics Committee of West China Hospital, Sichuan University. 1^*^10^6^ CT26 cells and 2^*^10^5^ CT26 cells were suspended in 100ul RPMI 1640 basal medium and injected subcutaneously and intraperitoneally, respectively, to establish subcutaneous tumor model and abdominal metastasis model. Animals were randomized into four groups before treatment: (A) NS: normal saline. (B) FG: fibrinogen + thrombin. (C) OXP. (D) OXP+FG. For subcutaneous tumor model, OXP 10 mg/kg together with fibrinogen 100μl + thrombin 100μl, fibrinogen 100μl + thrombin 100μl, OXP 10 mg/kg and NS 200μl, were given intratumorally 7 days after CT26 inoculation. For abdominal metastasis model, OXP 5 mg/kg together with fibrinogen 100μl + thrombin 100μl, fibrinogen 100μl + thrombin 100μl, OXP 5 mg/kg and NS 200μl, were given intraperitoneally 5 days after CT26 injection. Mice body weights and subcutaneous nodule sizes (length and width) were measured every 3 days. Tumor volume was calculated as the following formula: Tumor volume (mm^3^) = length^*^width^2*^0.5. Mice were euthanized when they presented cachexia or tumor surface started to ulcerate except that 3 mice from each group were euthanized three days after first treatment for early investigation on immune microenvironment. Ascites were collected by aspiration of a 5ml needle. Both abdominal nodules and subcutaneous tumors were weighted and prepared for histological investigation. Moreover, abdominal nodules were divided into > 3mm and < 3mm group according to diameter and counted by two independent investigators though naked eyes.

### Flow cytometry

Spleens were harvested, grinded, filtered and eventually suspended in PBS after red blood cells splitted with lysis buffer. Pellets of cells were dispersed in 100μl cold PBS and stained by 1μl of the following fluorochrome-conjugated antibodies: CD8, CD4, CD69 for 30min in dark and then washed with PBS. Intracellular Foxp3 staining was conducted with Foxp3/Transcription Factor Staining Buffer Set (affymetrix eBioscience) according to the product description. Stained cells were pretreated with cold PBS containing 5% FCS, then centrifuged pellets were dispersed in fixation/permeabilization buffer in 4°C dark atmosphere for 2h. Consequently, cells were washed and resuspended in 200μl permeabilization buffer. Afterward, FOXP3 antibodies were added and incubated in dark for 30 minutes. Cells were acquired and analyzed by NovoCyte flow cytometer (ACEA Biosciences, CA, USA).

### Ki_67_ and CD31 assay

For Ki_67_ and CD31 staining, 4μm paraffin embedded sections went through antigen retrieval and peroxidase inactivation. Following normal serum (ZSGB Bioscience, China) blockage, primary antibodies (goat anti-mouse Ki_67_ antibody and goat anti-mouse CD31 antibody, Servicebio, China) were added and incubated overnight at 4°C. Next, HRP-conjugated secondary antibodies (Servicebio, China) were added and incubated for 50minutes at 37°C. DAB was used to render color. A single microvessel was defined as a single cell with positive CD31. Cells with positive Ki_67_ staining represented cells with high proliferation activity. Specimens were observed in a light microscope (Ki_67_×200, CD31×100, Olympus, Japan). Ki_67_ Quantification was performed though calculating Ki_67_ positive cells percentage in five randomly selected fields at ×200 magnification. CD31 Quantification was performed though calculating CD31 positive cell amounts in five randomly selected fields at ×100 magnification

### TUNEL assay

To investigate the effect of OXP and FG on apoptosis of colon tumor, TUNEL staining is conducted in deparaffinized and hydrated tumor sections according to the manufacturer's instruction (Promega, USA). DAPI was used for nuclear staining. Specimens were observed in a fluorescence microscope (×100, Olympus, Japan). Quantification of apoptosis was performed though calculating TUNEL positive cells percentage in five randomly selected fields at ×100 magnification.

### ELISA of ascites

Supernatants of ascites were measured with mouse IFN-γ ELISA kit (affymetrix, eBioscience). All procedures were performed according to the product instruction. Briefly, 100μl/well standards and supernatants were added into capture antibody coated and blockage buffer blocked wells overnight, followed by incubation with detection antibody for 1h and Avidin-HRP for 30 minutes. Besides, washes with PBS containing 0.5% (v/v) Tween-20 solution (PBS-T) were conducted between each step. TMB substrate was added to color rendering in dark for 15minutes, which was finally ended by stop solution. Results were determined by a spectrophotometer at 450nm with a microplate reader. A standard curve was plotted to determine the quantity of IFN-γ in the test samples.

### Safety assessment

Organs including kidney, liver, lung, spleen, heart of mice were embedded in paraffin and cut into 4 μm-thick sections. HE staining was carried out as standard procedure dewaxing, hydration, Eosin staining, Haematoxylin staining and sealing. Sections were observed in a light microscope (×200, Olympus, Japan). Blood were collected after eyeball extraction and serums were harvested after overnight still placement at 4°C and measured for a few biochemical parameters (Hitachi, Japan).

### Statistical analyses

Graphic was performed with PRISM version 5.0 (GraphPad Software). Statistical analysis of multiple groups was performed with one-way analysis of variance (ANOVA). Statistical analysis between two groups was performed with Student's *t* test. Results were represented as mean ± standard error of the mean (SEM). A P-value of less than 0.05 was considered statistically significant for all experiments.
