# Correction: Improved anti-tumor efficacy via combination of oxaliplatin and fibrin glue in colorectal cancer

**DOI:** 10.18632/oncotarget.27605

**Published:** 2020-09-15

**Authors:** Yuzhu Hu, Ting Yu, Xiaoxiao Liu, Yihong He, Lihong Deng, Jiajuan Guo, Yuanqi Hua, Ting Luo, Xiang Gao

**Affiliations:** ^1^ Department of Head & Neck and Mammary Oncology and Department of Medical Oncology, Cancer Center, State Key Laboratory of Biotherapy, Laboratory of Molecular Diagnosis of Cancer, West China Hospital, West China Medical School, Sichuan University, Chengdu 610041, PR China; ^2^ Department of Neurosurgery and Institute of Neurosurgery, State Key Laboratory of Biotherapy/Collaborative Innovation Center for Biotherapy, West China Hospital, West China Medical School, Sichuan University, Chengdu 610041, PR China

**This article has been corrected:** During the assembly of Figure 8, two incorrect files were accidentally selected as the CD31 immunohistochemical images from NS and FG groups. The corrected Figure 8, using the proper images obtained from the original data, is shown below. The authors declare that these corrections do not change the results or conclusions of this paper.

Original article: Oncotarget. 2018; 9:2515–2526. 2515-2526. https://doi.org/10.18632/oncotarget.23507

**Figure 8:**
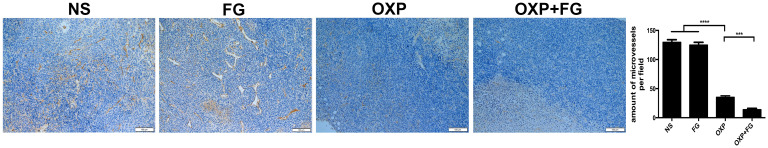
OXP and FG combination repressed tumor angiogenesis. CD31 staining can be used to label microvessel in tumor sections. Results showed reduced microvessel densities (MVD) of tumor cells in OXP group, compared with that in NS group and FG group (*P *< 0.0001, OXP group versus NS group and FG group.). There were less microvessels in OXP + FG group than that in OXP group (*P *< 0.001, OXP + FG group versus OXP group). Representative images of each group were showed above.

